# Two-Dimensional rGO-MoS_2_ Hybrid Additives for High-Performance Magnetorheological Fluid

**DOI:** 10.1038/s41598-018-30861-4

**Published:** 2018-08-23

**Authors:** Muhammad Taha Manzoor, Ji Eun Kim, Jung Hwan Jung, Chulhee Han, Seung-Bok Choi, Il-Kwon Oh

**Affiliations:** 1 Creative Research Initiative Center for Functionally Antagonistic Nano-Engineering, Department of Mechanical Engineering, Korea Advanced Institute of Science and Technology (KAIST), 291 Daehak-ro, Yuseong-gu, Daejeon, 34141 Republic of Korea; 2 LG Chem, Ltd., 30 Magokjungang 10-ro, Gangseo-gu, Seoul, Republic of Korea; 3 Smart Structures and Systems Laboratory, Department of Mechanical Engineering, Inha University, Incheon, 402-751 Republic of Korea

## Abstract

Magnetorheological fluids (MRF) that undergo a change in their viscoelastic properties under the magnetic fields have been considered as one of most important smart functional materials for vibration dampers and shock absorbers in several engineering applications. However, the use of magnetorheological fluids in practical applications has been limited by poor sedimentation ratio and on-state yield stress. Herein, we report hybrid rGO-MoS_2_ additives for a high-performance magnetorheological fluid. Two different kinds of hybrid additives, which are called non-magnetic rGO-MoS_2_ and magnetic Fe-rGO-MoS_2_, were synthesized by using a hydrothermal method. The rGO-MoS_2_ added suspensions remained stable for the first 90 min whereas the CIP MRFs settled down quickly (65%) in the first 10 minutes. The Fe-rGO-MoS_2_ additives showed a 24% higher on-state shear stress as compared to CIP MRFs. On the other hand, an increase of 60% in the on-state yield stress for Fe-rGO-MoS_2_ MRF can be attributed to the gap-filling by the hybrid additives during columnar-structure formation. Among two-dimensional (2D) materials, Molybdenum Disulphide (MoS_2_) is a member of transition metal dichalcogenides (TMDCs), traditionally used as solid lubricant, while reduced graphene-oxide (rGO) is a well-known 2D material with supreme mechanical properties. We believe that this study will blaze the new way for developing a high-performance magnetorheological fluids based on various 2D material hybrids.

## Introduction

A branch of rheology that studies the flow and deformation of the materials upon the application of an applied magnetic field is known as magnetorheology^1^. Magnetorheological fluids (MRF) can be defined as the non-Newtonian fluids that undergo a change in their viscoelastic properties when the magnetic field is applied^1^. In reality, an MRF consists of three main components, i.e., a fluid medium (synthetic oil), magnetisable particles (carbonyl iron powder) and additives^2^. Additives are added to improve some particular rheological property. In the absence of the magnetic field, the iron particles remain suspended in the oil and the mixture behaves like a normal liquid. When the magnetic field is applied, the iron particles align themselves in the form of columnar structures^1–3^. As a result of these strong structures, the mixture becomes highly viscous. This classic feature of a rapid change in the viscosity and yield stress can be utilized in the engineering applications related to vibration dampers and shock absorbers in automotive vehicles and home appliances^1^.

Many researchers have contributed towards the enhancement of the performances of MRF. Before going into details, it seems necessary to mention the key performance parameters for the MRF that include: on-state yield stress, sedimentation ratio and on-state shear stress^1–3^. The above-mentioned properties are pre-dominantly dependent on the magnetic/non-magnetic nature, the size and the shape of the additives used in the fluid. Hence, researchers have utilized various kinds of additives to investigate their effects on MRF properties^1^. To mention a few, Ulicny stated that non-magnetic particles can be used to improve the performance of MRFs^4^. Bombard *et al*.^5^ also recently reported the effect of using non-magnetic goethite nano-fiber particles as additives in the MRF. In addition, they noted that the shape of the particles plays a major role in the enhancement of the performance as compared to its magnetic properties. In one study, magnetic (Fe_3_O_4_) and non-magnetic (Fe_2_CO_4_. 2 H_2_O) rods were employed as additives and demonstrated to be fruitful for the sedimentation delay^6^. Nagashima *et al*. observed an enhancement in the response of magneto-elastomers by introducing non-magnetic zinc oxide particles^7^. Levin *et al*.^8^ reports an increase in the viscous stress by the addition of non-magnetic abrasive additives. On the other hand, it is reported by Ngatu *et al*. that magnetizable (spherical) additives only delayed the sedimentation, but had no significant effect on the on-state yield stress^9^. Moreover, the size of the additives in micro and nano levels has shown a remarkable effect on the properties as well. Submicron iron micro wires^10^ and nano-sized non-magnetic^5^ and superparamagnetic^11^ additives have displayed significant improvements. In short, the literature review reveals that depending on the nature of a particular additive, there might be a trade-off between the MRF properties; thus, a thorough investigation is still required in order to comprehensively understand the effects of non-magnetic and magnetic additives on the MRF properties.

In addition, two-dimensional (2D) materials have been investigated thoroughly during the past decade. The obvious reason behind that quest is their remarkable mechanical, electrical, thermal and optical properties^12–15^. Among 2D materials, the transition metal dichalcogenides (TMDs) are of special interest because they present a vast variety of 2D materials with surprisingly different properties^16,17^. MoS_2_ is one of the TMDs that can be converted to 2D material with sonication^18^. MoS_2_ is inherently non-magnetic and is reported to be a semi-conductor (band gap 1.8 eV)^19^. Although, MoS_2_ has been widely used as solid lubricant in auto-mobile industry. To the best of our knowledge, the effect of MoS_2_ as an additive on the rheological properties of MRF has not been investigated extensively, yet.

Similarly, reduced graphene oxide (rGO) is another important 2D material with a non-magnetic property^20^. When graphene oxide (GO) is reduced, partial restoration of the graphitic structure is achieved^21–26^, which is favorable for industrial applications^27–29^. Choi *et al*.^30^ reported positive effects of graphene oxide on the shear stress and sedimentation rate. However, they recorded that increasing the additive ratio had a subdue effect on the shear stress property.

The hybrid structures offer an advantage to utilize the favorable characteristics of each component. Nowadays, the research focus is on the synthesis of composites having better properties than their respective components^31^. Some studies report^32^ that the hybrid structures had superior properties than any of its individual component. The tweakable properties of hybrid nano-materials can be advantageous in the field of MRF to avoid the trade-offs between sedimentation ratio and magnetic-field dependent properties. Therefore, in order to investigate the effects of hybrid structures as additives, and, to combat the problem of sedimentation, low yield and shear stress, we propose the use of rGO-MoS_2_ and iron decorated rGO-MoS_2_ hybrid additives.

In the present study, our aim is to introduce non-magnetic rGO-MoS_2_ and magnetic iron nano particles decorated rGO-MoS_2_ (Fe-rGO-MoS_2_) additives and investigate their effects on MRF performances. The study evolves in a gradual manner. Firstly, the effect of commercially bought MoS_2_ additive is investigated by measuring the rheological properties. MoS_2_ shows promising effect on the sedimentation rate. Later, hybrid additive rGO-MoS_2_ is synthesized and added to the MRF. The results are compared for both the non-magnetic additives. In the next phase, iron nanoparticle decoration (Fe-rGO-MoS_2_) is done in order to impart the magnetic property to the hybrid additive. Finally, all the results are compared, and it is noted that the rGO-MoS_2_ hybrid additive displayed the best performance, among the additives used in the present study, for sedimentation property. Shear stress was improved but the effect on the yield stress was small. On the other hand, Fe-rGO-MoS_2_ enhanced the yield and shear stress properties significantly, along with a sedimentation rate comparable with the commercial MoS_2_. The iron decoration process was very simple while providing considerable improvement in the rheological properties. Even a slight magnetism gave significant results. The technical novelty lies in the fact that the hybrid nature may help in overcoming the trade-offs between the key MRF properties. Moreover, easy tweaking of the hybrid 2D materials may give the power to design the MRF additives according to the particular application. Therefore, we report that a magnetic iron decorated rGO-MoS_2_ hybrid additive can be a promising choice for enhancing the magneto-rheological properties.

## Experimental Procedures

### Materials and Method

The carbonyl iron powder (CI) was purchased from BASF SE, Germany (average size 3–4 µm, density 7.86 g/cm^3^, EW grade) while the commercial MoS_2_ powder was obtained from Sigma Aldrich (average size <2 µm, density 5.06 g/cm^3^). Chevron Phillips, USA provided the Synfluid PAO 2 cSt synthetic oil (viscosity 5.1 at 40 °C, density 0.79 g/cm^3^). All these materials were used as purchased without any further treatment. The improved Hummer’s method was used to synthesize graphene oxide (GO). The chemicals used for the synthesis of GO, such as, graphite flakes, Potassium permanganate (KMnO_4_), Sulphuric acid (H_2_SO_4_) and Phosphoric acid (H_3_PO_4_) were purchased from Sigma Aldrich (Germany). Thiourea and ammonium molybdate tetrahydrate ((NH_4_)_6_Mo_7_O_24_.4H_2_O) used for the synthesis of the rGO-MoS_2_ hybrid additives were also obtained from Sigma Aldrich. The iron decoration was achieved in a hydrothermal process by using Iron (II, III) oxide (Fe_3_O_4_) nano-particles bought from Daejung, Korea.

### Synthesis of Graphene Oxide

The improved Hummer’s method was utilized because it offers better efficiency of the oxidation process. Moreover, this method is safer than other methods as it does not generate any toxic gases with an easy control on temperature^21^. Thus, considering the industrial application of the additives reported in this study, the improved Hummer’s method was chosen. The procedure followed is the same as narrated by Marcano *et al*.^21^. A 1:6 ratio of graphite flakes and KMnO_4_ was used along with 9:1 ratio of H_2_SO_4_ and H_3_PO_4_. The mixture was left for stirring at 50 °C for 24 hours. After stirring for 24 hours, the mixture was poured onto the ice and a few milliliters of H_2_O_2_ were added. The mixture was kept in a beaker and decanted after regular intervals. When the pH was noted to be neutral, centrifugation was done to remove any un-exfoliated graphite. GO obtained in this step was later used for synthesizing rGO/MoS_2_ hybrid additives.

### Sythesis of rGO-MoS_2_ Hybrid Additive

After obtaining the GO in pervious step, MoS_2_ was grown on the surface of the rGO by using hydrothermal process^33^. The precursors used for the process were thiourea and ammonium molybadte tetrahydrate (1:13). Both of the precursors were dissolved in DI water and later mixed with GO. The whole mixture was then stirred for 30 minutes. Teflon lining 350 ml reactor was used and kept in oven at 200 °C for 24 hours. Upon the completion of the reaction, the reactor was removed from the oven and allowed to cool down under room conditions. The mixture was washed with DI water and ethanol by using vacuum filtration. Later, the purified hybrid material was dried in an oven. These rGO-MOS_2_ additives were used in the next step for iron decoration.

### Fe-rGO-MoS_2_ Hybrid Additive Synthesis

**Figure 1:**
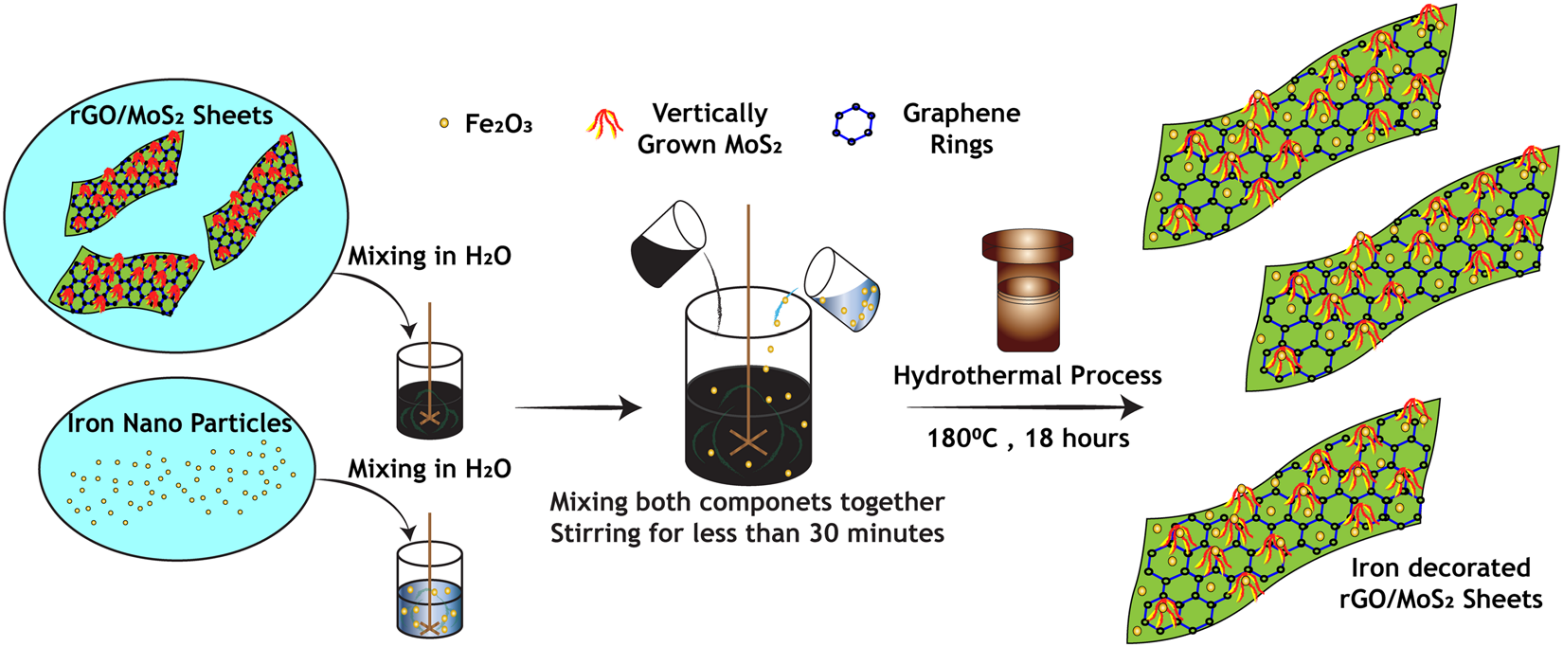
Schematic diagram demonstrating the step-wise synthesis procedure employed for iron decorated rGO/MoS_2_ hybrid additives.

The rGO-MoS_2_ obtained in the last step was used for the iron decoration process. The procedure was very simple and straightforward involving the mixing of Fe_3_O_4_ nano-particles with rGO-MoS_2_ (1:4 and 1:2)^34^. The mixture was again transferred to Teflon lining reactor and kept in oven at 180 °C for 18 hours. The reactor was removed from the oven after 18 hours, cooled, washed, filtered and dried in oven at 60 °C. The procedure is schematically represented in Fig. 1.

### Preparation of MR Fluids

The MRF preparation required efficient mixing of CI particles with additive materials in PAO. High volume ratios (45 vol%) were used which posed the problem of high viscosity, agglomeration and poor mixing. Therefore, n-hexane was used along with PAO (1:1) to lower the viscosity of the mixture in mixing phase. n-hexane is a low boiling liquid; hence, it was easily evaporated after the proper mixing was achieved. This technique provided an easy and efficient way to mix solid particles in PAO without agglomeration problems. A mechanical homogenizer was used to mix the solid particles in PAO/n-hexane followed by the removal of n-hexane in oven at 70 °C.

## Materials Characterization

### Chemical Characterization

**Figure 2:**
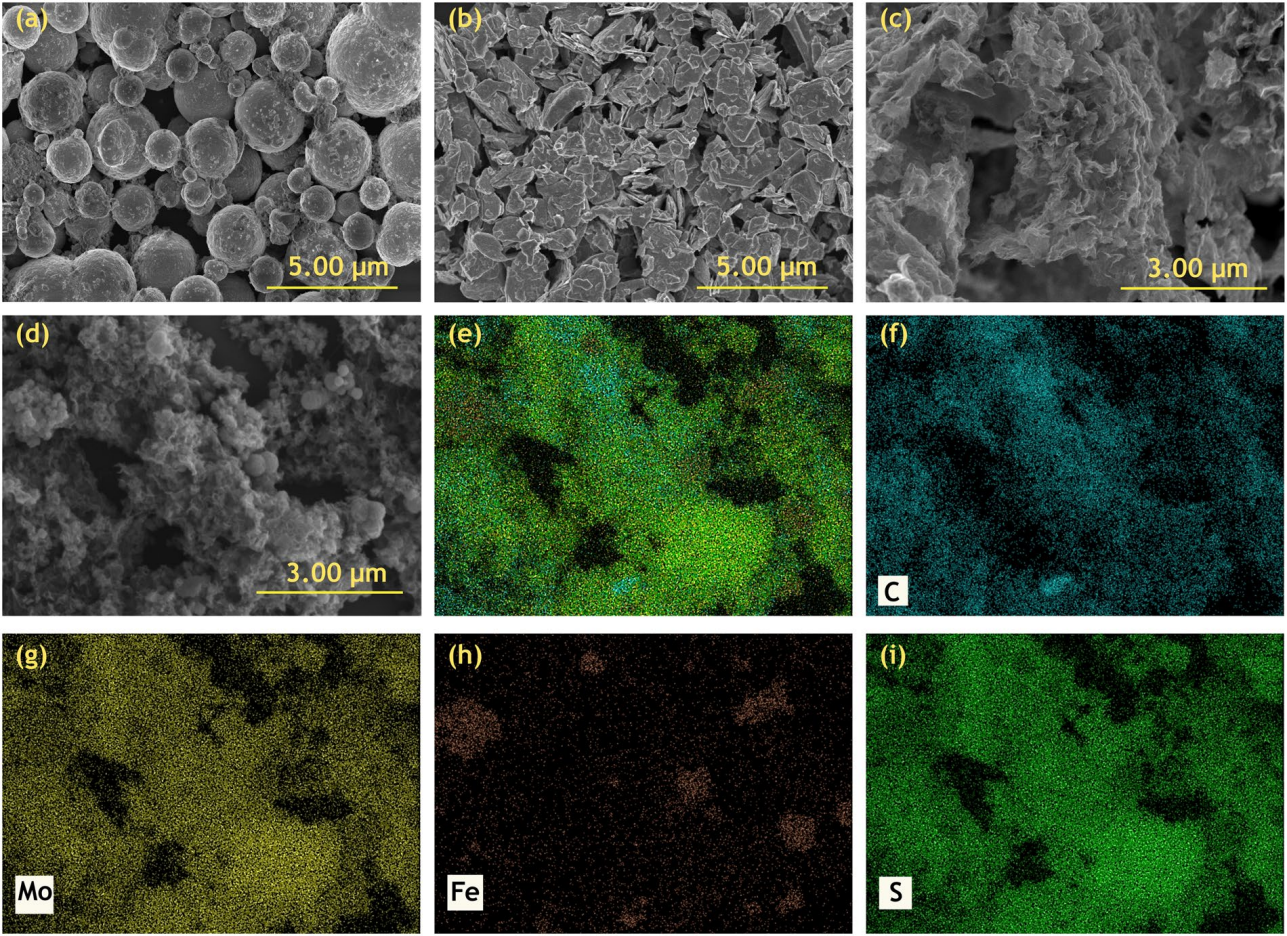
SEM images (**a**) commercial carbonyl iron powder; (**b**) commercial MoS_2_; (**c**) rGO-MoS_2_ and (**d**) Iron decorated rGO-MoS_2_ (**e**) EDS mapping of Fe-rGO-MoS_2_ (overlay image) (**f**–**i**) shows the mapping for Carbon, Molybdenum, Iron and Sulphur respectively.

In order to investigate the morphology of the particles used in the MRF, scanning electron microscopy (SEM) was employed. In Fig. 2a, commercially bought CI particles are shown having spherical morphology. Figure 2b presents the plate like morphology of sonicated commercial MoS_2_ powder. The MoS_2_ grown on the rGO sheets can be observed in Fig. 2c. There is a very little free grown MoS_2_ while vertically grown MoS_2_ on rGO sheets can be seen clearly. The iron decorated sheets can be observed in Fig. 2d, iron nano-particles cluster together to form small spheres like shapes, there presence can be confirmed by EDS mapping of Iron in Fig. 2h. Other important elements such as C, Mo and S are also visible in EDS mapping (Fig. 2e–i). The SEM images and EDS mapping confirmed the synthesis of good morphological hybrid additives.

**Figure 3:**
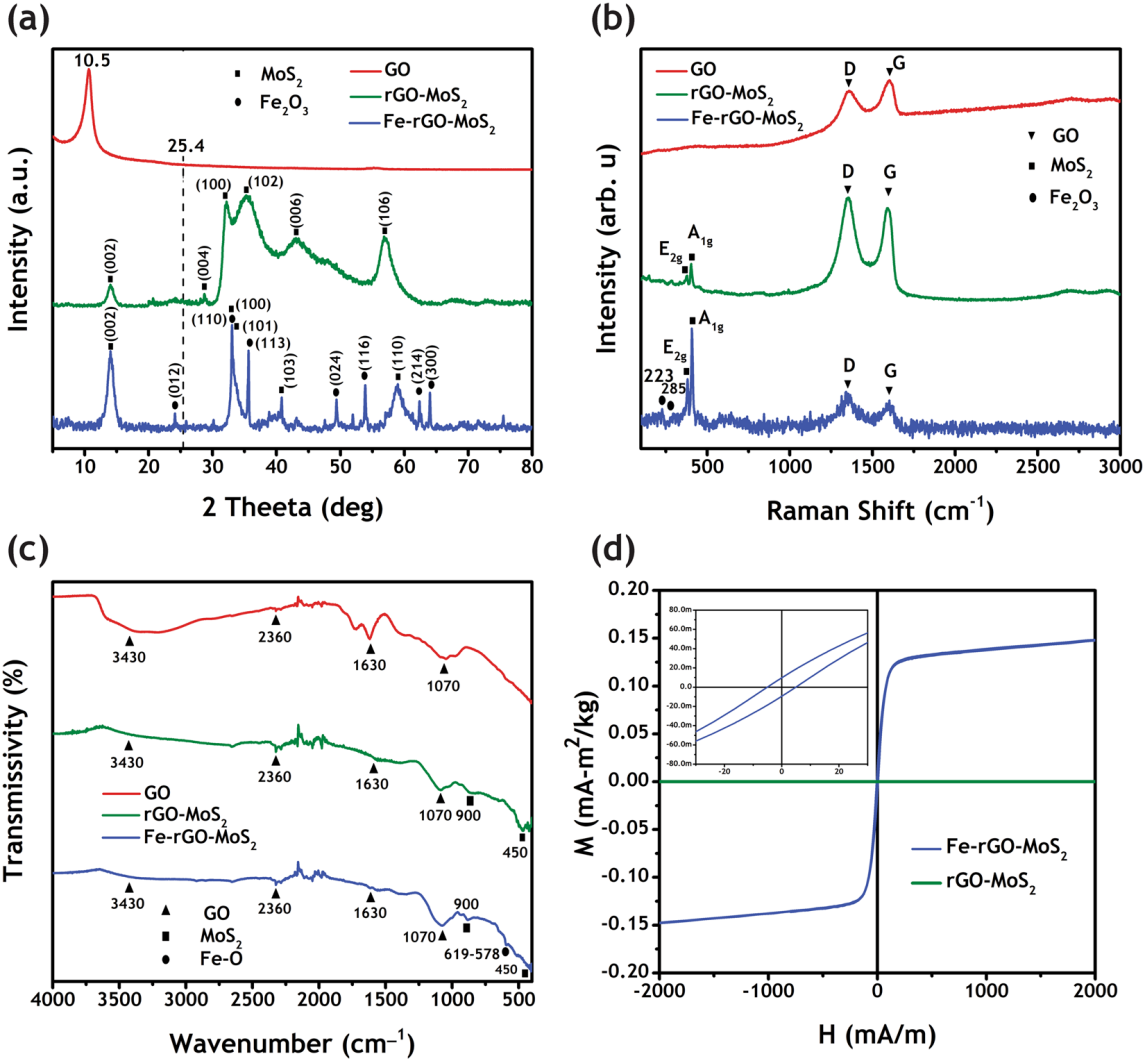
Material characterization for GO, rGO-MoS_2_ and Fe-rGO-MoS_2_ (**a**) XRD patterns; (**b**) Raman Spectroscopy and (**c**) FT-IR analysis (**d**) The magnetization curves for Fe-rGO-MoS_2_ and rGO.

Figure 3a shows the XRD pattern for the synthesized additives. A clear peak can be seen at 10.5° which is the characteristic peak for GO. This peak reveals that the graphite flakes were oxidized and exfoliated (spacing increased from 0.34 nm to 0.82 nm)^34^. The pattern for rGO-MoS_2_ demonstrates an absence of peak at 10.5° and presence of a broad peak at 25.4° indicating that GO was reduced successfully. Moreover, it also indirectly indicates that there was a minor re-stacking between the rGO sheets and it was well hybridized with MoS_2_. The other peaks (attributed to (002), (004), (100), (102), (006) and (106) planes) matched well with the MoS_2_ XRD patterns^33,35^. Finally, the pattern for Fe-rGO-MoS_2_ is shown at the bottom. Again, it can be seen that the GO was reduced and iron particles were decorated (a series of characteristic peaks can be indexed to (012), (110), (113), (024), (116), (214) and (300) planes of Fe_3_O_4_^36^).

Figure 3b represents the Raman spectroscopy results. The characteristic D band (1,355 cm^−1^) and G band (1,603 cm^−1^) with intensity ratio (I_d_/I_c_) of 0.9 confirm the synthesis of GO^35^. For MoS_2_, E^2g^ and A^1g^ bands are observed at 361 cm^−1^ and 420 cm^−1^, respectively^37^. Along with that the intensity ratio (I_d_/I_c_) increased from 0.9 to 1.01 indicating reduction of GO^35^. The bottom curve shows the Raman pattern of Fe-rGO-MoS_2_. The peaks at 223 cm^−1^ and 285 cm^−1^ indicated the presence of iron nano-particles^38^. The intensity ratio was noted to be 1.03 indicting the removal oxygen^36^. Sharp peaks of E^2g^ and A^1g^ bands proved that MoS_2_ was still present on rGO sheets and confirmed the successful synthesis of hybrid nano-composite.

In order to investigate the nature of chemical bonds, FT-IR analysis was utilized. In Fig. 3c, the peaks at 1,070 cm^−1^, 1,630 cm^−1^, 2,360 cm^−1^ and 3,430 cm^−1^ can be attributed to the stretching of C-O-C, C=C, C=O and –OH bonds, respectively^36^. The C=O and -OH peaks were not observed to be sharp, while C=C and C-O-C peaks were sharp. For MoS_2_, the peak at 450 cm^−1^ represents Mo-S stretching, while S-S peak was observed at 900 cm^−1^ ^35^. On the other hand, the peak intensity for C=C bonds was reduced while the peaks corresponding to C=O and C-O-C bonds were observed to be sharper than before. Weakening of the peak at 1,630 cm^−1^ indicates the reduction of GO. In the case of Fe-rGO-MoS_2,_ peaks between 619–578 cm^−1^ represent the Fe-O bond stretching^36^.

### Magnetic Property

In the present study, two different kinds of additives were employed: magnetic and non-magnetic. Therefore, magnetic properties of dry rGO-MoS_2_ and Fe-rGO-MoS_2_ powders were investigated by using Vibrating Sample Magnetometer (VSM) at room temperature conditions. Figure 3d shows that the rGO-MoS_2_ additive was non-magnetic and did not show any magnetism. Two different ratios for iron decoration were employed (Fe_3_O_4_:rGO-MoS_2_, 1:4 and 1:2). The low loading displayed a minor magnetic effect and showed hysteresis as well in Fig. 3d. On the other hand, a stronger magnetic effect was observed for higher loading ratio as shown in Fig. S2a. The sedimentation rate tests revealed that the higher iron loadings produced a negative effect due to its high weight (Fig. S2b). Thus, additives with lower loading were used for further investigation. Considering the industrial applications, this choice is economically feasible as well. The inset figure shows a zoomed image around the origin. The magnetization loops were observed to be very narrow, which means that these materials respond as soft materials. The soft magnetic nature is an important criterion for MRF. In MRF, the solid particles must be capable of a quick reversible change (magnetization and demagnetization) when a magnetic field is applied^39,40^. Therefore, the additives used in this study could be capable of enhancing the rheological properties, effectively.

## Results of MRF Performances

### Sedimentation Rate

**Figure 4:**
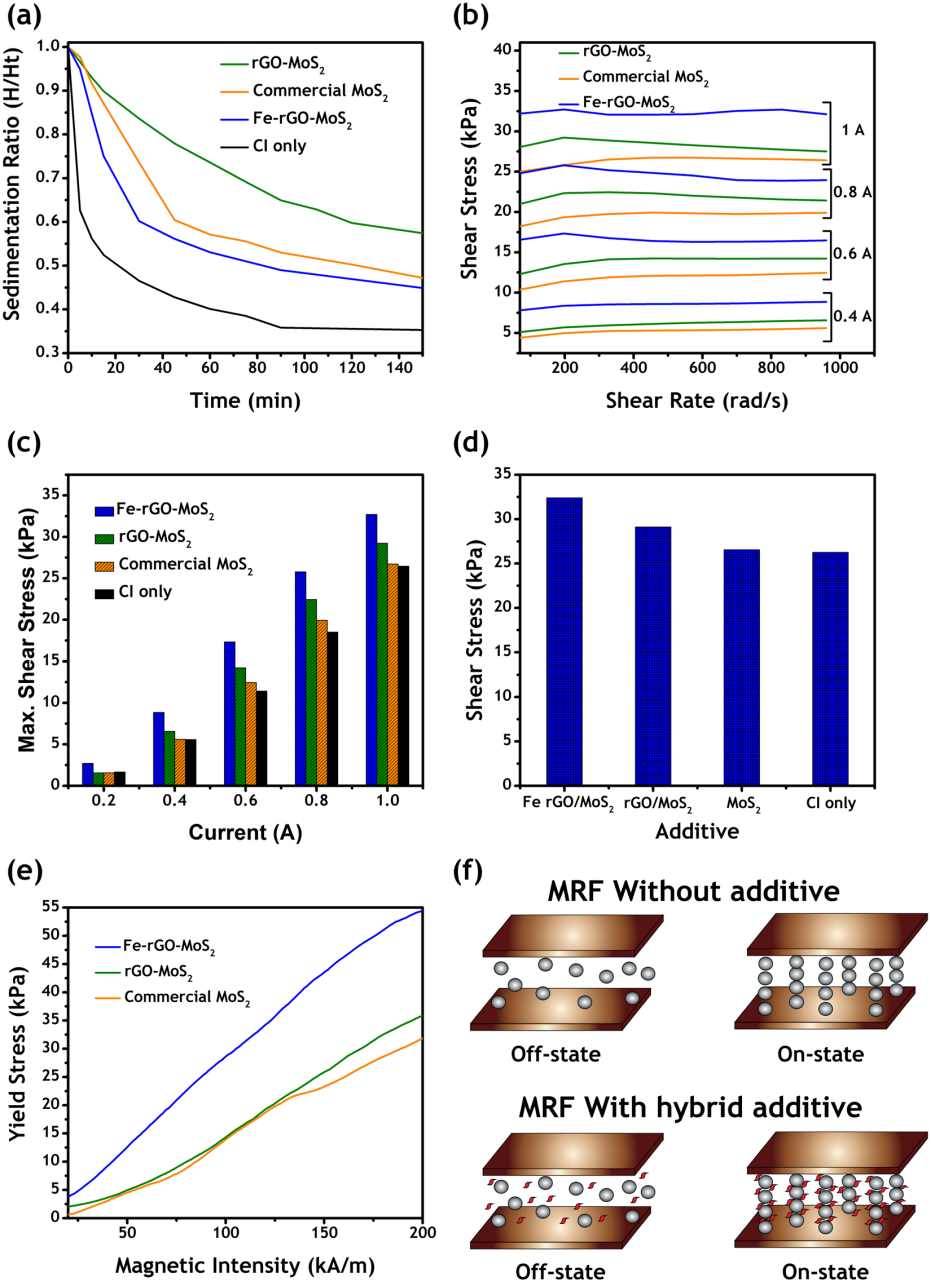
Rheological properties (**a**) Sedimentation rate data; (**b**) shear stress vs shear rate at different currents; (**c**) Comparison data for maximum shear stress at different currents; (**d**) Comparison data for difference between on state and off state shear stress for each additive i.e. the maximum possible change in the shear stress; (**e**) Yield stress vs magnetic intensity; (**f**) schematic for the mechanism responsible for high shear and yield stresses for magnetic hybrid additives.

In MRFs, one of the important properties is the sedimentation rate. Due to the density mismatch between the solid particles and the oil medium, the solid particles tend to settle down as the mud at the bottom^1,5^. In order to measure the sedimentation rate quantitatively, a simple experiment was designed. A narrow graduated glass flask (10 ml) was selected and filled with the MRF. The phase boundary between the supernatant oil and the concentrated suspension was observed with respect to time until it attained a steady state. The MRFs were diluted by adding 50% additional PAO so that the interface travel rate is fast and fine results are obtained in lesser time. In order to calculate the sedimentation rate, the following simple equation (1) was used:

1 $$SR=\frac{{H}_{S}\,}{{H}_{T}}$$

where $SR$, ${H}_{S}$ and ${H}_{T}$ are sedimentation ratio, height of the sediment, and total height of the entire suspension, respectively. The additive volume ratios (3 vol%), total volume ratio (45 vol%) and the amount of PAO for dilution was kept constant in each test (Fig. 4a). First, the CI MRF without any additives was tested for sedimentation property. It was noted that the sedimentation rate was very fast in the first 10 minutes (65% settled) and attained a steady state after 90 minutes.

In the next stage, the commercial MoS_2_ powder was used as an additive in order to investigate its effects on the sedimentation ratio. It was observed that the sticky and grease-like nature of the MoS_2_ inside the fluid caused a significant reduction in the sedimentation rate (Fig. 4a). Sedimentation was relatively quick in the first 40 minutes, but relatively slow when compared to the CIP MRF. This unique observation hints at that the MoS_2_ can be an ideal additive for the sedimentation reduction. Along with the sedimentation property, the MoS_2_ is an excellent solid lubricant. Hence, it can be used to avoid any damage to the internal parts of the MRF based systems.

The volume ratios of the commercial MoS_2_ additives were changed and it was noted that increasing the concentration of MoS_2_ reduces the sedimentation rate (Fig. S3a). On the other hand, the on-state shear stress properties were effected negatively (Fig. S3b). The reason behind this reduction in on-state shear stress is that the total vol% was fixed to 45 vol%. Therefore, an increase in additive ratio means a decrease in CIP ratio. The major magneto-rheological effect is governed by the CIP (large sized magnetizable particles). If there is a critically low amount of CIP, then the fluid could not respond to the magnetic fields effectively, which may lead to detrimental effects on on-state shear stress properties. Thus, considering this limitation a volume ratio of 3% was selected for further investigation and comparisons.

Two kinds of hybrid additives were utilized in the sedimentation test: non-magnetic (rGO-MoS_2_) and magnetic (Fe-rGO-MoS_2_). The rGO-MoS_2_ additives displayed the best performance among all the studied MRFs. The suspension remained relatively stable (small slope of line) for the first 90 minutes. The Fe-rGO-MoS_2_ additives showed better performance than the CI only samples and closer to the commercial MoS_2_ additives. As discussed before, increasing the loading level of iron nano-particles on rGO-MoS_2_ caused the additives to become heavier; therefore, in order to avoid any detrimental effects on the sedimentation rate, a controlled amount of iron particles was chosen with a ratio of 1:4 (for Fe_3_O_4_: rGO-MoS_2_). For the first half hour, the additive showed a quick sedimentation, but as the time passed, it became stable and matched the commercial MoS_2_ rate. These sedimentation tests reveal that if high on-state rheological properties are not desired then rGO-MoS_2_ hybrid additive can be a potential candidate for countering the problem of high sedimentation rate.

### Magnetorheological Effect

Figure 4b represents the data for the on-state shear stress with changing shear rate at different amperes. As the current, as an index for magnetic field intensity, increased from 0.4 A to 1 A, the shear stress also increased for all the MRF samples. The magnetic field intensity can be related to the applied current by equation (2) as shown below:

2 $$H=149.33\,{I}^{4}-288\,{I}^{3}+110.67\,{I}^{2}+100\,I$$

where H and I are the magnetic field intensity (kA/m) and applied current (A), respectively. Thus, a magnetic field intensity of 72, 64.5, 57, 43.1 and 22.3 kA/m was obtained by applying 1.0, 0.8, 0.6, 0.4 and 0.2 A, respectively. The rGO-MoS_2_ samples displayed a better performance than the commercial MoS_2_ powder, which indicates that the hybrid nature of the additive caused the enhancement even without the magnetic effect (Fig. 4c). In order to investigate the effects of magnetism of the additives, Fe-rGO-MoS_2_ samples were tested. It was observed that the magnetic nature of the additives played a significant role in the enhancement of the shear stress. Even slight magnetism (Fig. 3d) dramatically improved the shear stress response as compared to the non-magnetic rGO-MoS_2_ and commercial MoS_2_ powder (Fig. 4c). At 0.8 A, the Fe-rGO-MoS_2_ additives showed an equivalent performance as the commercial MoS_2_ at 1 A for a shear rate till 200 rad/s. The Fe-rGO-MoS_2_ based MRFs out-performed all other MRFs at each given condition. Figure 4d represents the difference between maximum on-state and off-state shear stress. It is a direct measure of the range in which the particular MRF can work. A larger value means that the rheological properties can be varied in a vast range. Fe-rGO-MoS_2_ has the largest range among the studied additives (23% higher than CI only MRF). The rGO-MoS_2_ also had better range than MoS_2_ and CI only MRF samples.

The yield stress is another important property that was needed to evaluate the MRF performance. The results for the yield stress with changing magnetic intensity are summarized in Fig. 4e. It can be seen that the non-magnetic rGO-MoS_2_ additives performed better than the commercial MoS_2_ at all magnetic intensities but the significant enhancement was observed at higher intensities (above 130 kA/m). Still the difference was not very big between the two additives; maximum value of 36 kPA and 31 kPA for rGO-MoS_2_ and commercial MoS_2,_ respectively. From this observation, it is concluded that rGO-MoS_2_ additive performs better in shear stress than in yield stress. Therefore, in order to improve the yield stress, magnetism was imparted to the rGO-MoS_2_ additives by iron decoration. The slight iron decoration improved the yield stress response significantly, maximum value of 54 kPa as compared to the 36 kPA (rGO-MoS_2_) and 31kPa (commercial MoS_2_).

The results of shear stress and yield stress clearly indicate that magnetism of the additives play a pivotal role. The hybrid additives take the advantage of the supreme properties of their parts and enhance the MR effect. As supported by the XRD results (Fig. 3a), there was a minor re-stacking between the rGO sheets due to the presence of MoS_2_. The lesser re-stacking means that the sheets had more surface area^32^. On the other hand, by using the hybrid structure, the agglomeration of MoS_2_ sheets was avoided, which lead to the utilization of superior mechanical properties of the 2D structures^32^. Thus, for rGO-MoS_2_ additives an enhancement in the shear stress property was observed as compared to MoS_2_. Furthermore, the well spread sheets might have supported the CI particles in the fluid medium. Moreover, the low density and sticky nature of the MoS_2_ could have helped in the process. Thereby, producing a combined effect which reduced the settling down rate of particles.

The non-magnetic nature of the rGO-MoS_2_ additives, negatively affected the yield stress response. The problem was addressed by the iron decoration of rGO-MoS_2_ additives. When magnetic field is applied, the magnetic nano-additives will try to align themselves along with the micro CI particles. These nano-particles can fill the gaps between adjacent spheres. Thereby, producing stronger columnar structures; thus achieving higher on-state yield and shear stress (see Fig. 4f for the schematic representation)^30^. Our results on yield stress and shear stress strongly support the above-mentioned phenomena (60% higher yield and 24% higher shear stress than CIP MRF). Although, non-magnetic particles might also fill these gaps but it would not be able to form any strong columnar chains structures (as evident by a small increase (10%) in the on-state shear of rGO/MoS_2_ MRF as compared to CI only MRF). In summary, magnetism of the hybrid additives could be advantageous in enhancing the on-state rheological properties. Hence, it can be inferred that the Fe-rGO-MoS_2_ hybrid additives are promising candidate for the improvement of MRF responses.

## Conclusion

In conclusion, the MRF can respond to the magnetic fields as smart fluids. Their rheological properties depend on the types of solid particles used in the preparation. Fast sedimentation rates hinder the practical use of the MRF. In order to cater for this disadvantage, various types of additives were employed in previous studies but almost each of them came with a tradeoff between the sedimentation rate and other field dependent properties. This situation demands for a class of additives with tweakable rheological properties. Therefore, in this study we have reported hybrid two-dimensional additives for MRF applications.

First, the commercially bought MoS_2_ powder was used and sedimentation rates were measured indicating that MoS_2_ caused a decrease in sedimentation rate. Later, Hummer’s method was used to synthesize GO. The GO was reduced and MoS_2_ precursors were used to grow MoS_2_ on its surface. Finally, rGO-MoS_2_ samples were decorated with iron, in a hydrothermal process, to impart magnetism. SEM images and elemental mapping confirmed the morphology of the synthesized materials. The sedimentation rate experiments were performed by using a 10 ml flask; allowing the MRF to settle down meanwhile noting the sediment height. Material characterization was done using established techniques like XRD, Raman spectroscopy and FT-IR. The results confirmed the success of the hybridization process. VSM technique was used to confirm the magnetism of the Fe-rGO-MoS_2_ powders.

The rGO-MoS_2_ additives showed better sedimentation rate performance as compared to all other additives used in this study. There was an increase in the shear stress property as well; however, there was a slight improvement in yield stress property. The iron decorated samples displayed a dominating effect on both the on-state yield and shear stress properties. The reason behind this performance is the increased surface area (due to hybrid structure) and gap-filling by the magnetic nano-hybrid additives. In summary, the hybrid additives offer a wide variety of unique properties, which are better than their individual parts. These properties can be tweaked easily and could be advantageous for further enhancement of rheological properties. In future, more kinds of hybrid structure with different morphologies and 2D material combinations can be designed for particular MRF applications.

## Electronic supplementary material

Supplementary Material
