# Supplementary material for: Two-Dimensional rGO-MoS2 Hybrid Additives for High-Performance Magnetorheological Fluid
Source: Sci Rep. 2018 Aug 23;8:12672. doi: 10.1038/s41598-018-30861-4 (PMC6107629; doi:10.1038/s41598-018-30861-4)
Supplement: Supplementary file 1 — Supplementary Material [file 41598_2018_30861_MOESM1_ESM.pdf]

Supplementary Information

Title

Two-Dimensional rGO-MoS_2 Hybrid Additives for High-Performance Magnetorheological Fluid

Muhammad Taha Manzoor[1], *Ji Eun Kim*[1,2], *Jung Hwan Jung*[1], *Chulhee Han*[3], *Seung-Bok Choi*[3,*] *and Il-Kwon Oh*[1,*]

[1] Creative Research Initiative Center for Functionally Antagonistic Nano-Engineering, Department of Mechanical Engineering, Korea Advanced Institute of Science and Technology (KAIST), 291 Daehak-ro, Yuseong-gu, Daejeon 34141, Republic of Korea
Muhammad Taha Manzoor, Ji Eun Kim, Jung Hwan Jung & Il-Kwon Oh

[2] LG Chem, Ltd., 30 Magokjungang 10-ro, Gangseo-gu, Seoul, Republic of Korea

Dr Ji-Eun Kim

[3] Smart Structures and Systems Laboratory, Department of Mechanical Engineering, Inha University, Incheon 402-751, Republic of Korea
Chulhee Han & Seung-Bok Choi

* Corresponding Authors: Il-Kwon Oh (Tel.: +82-42-350-1520, E-mail: ikoh@kaist.ac.kr)
and Seung-Bok Choi (Tel: +82-32-860-7319, E-mail: seungbok@inha.ac.kr)

Keywords: Magnetorheological fluids; Molybdenum disulphide; Reduced graphene oxide; Hybrid two-dimensional materials

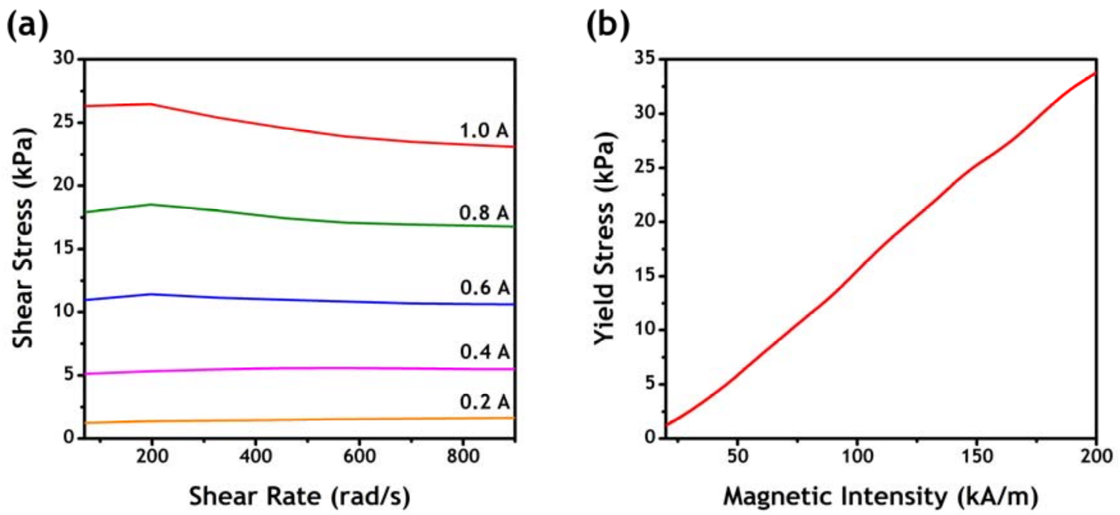

Figure S1: CI only MRF data (a) Shear Stress at changing shear rate and applied electric currents (b) Yield stress for changing magnetic field intensity

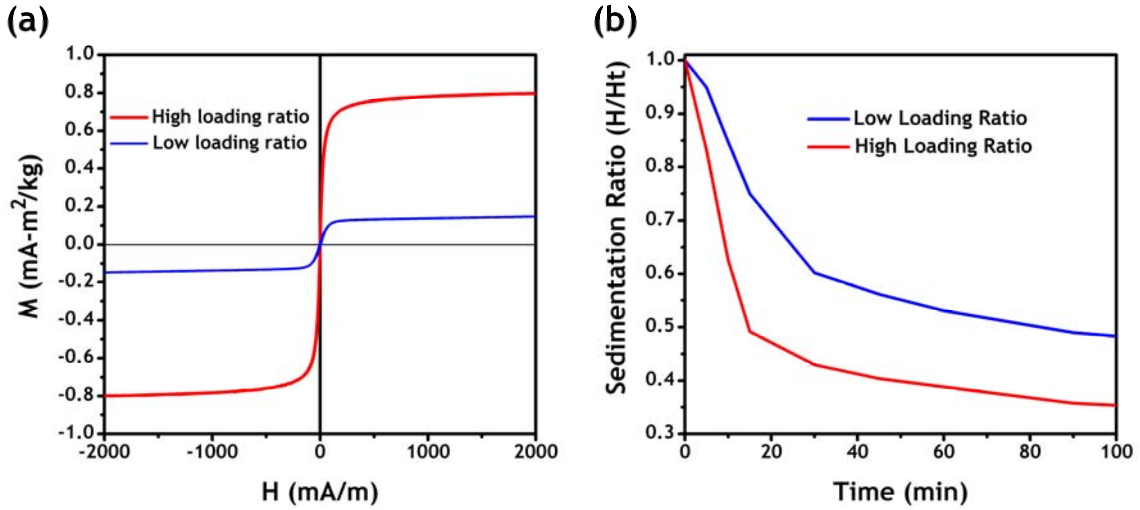

Figure S2: Different synthesis ratio used for Fe-rGO-MoS_2 (a) Magnetization curves for two different ratios used for Fe-rGO-MoS_2 synthesis; high loading shows stronger magnetism. (b) Sedimentation rate for both samples; due to heavy iron particles loading the high weight additives settled down quickly.

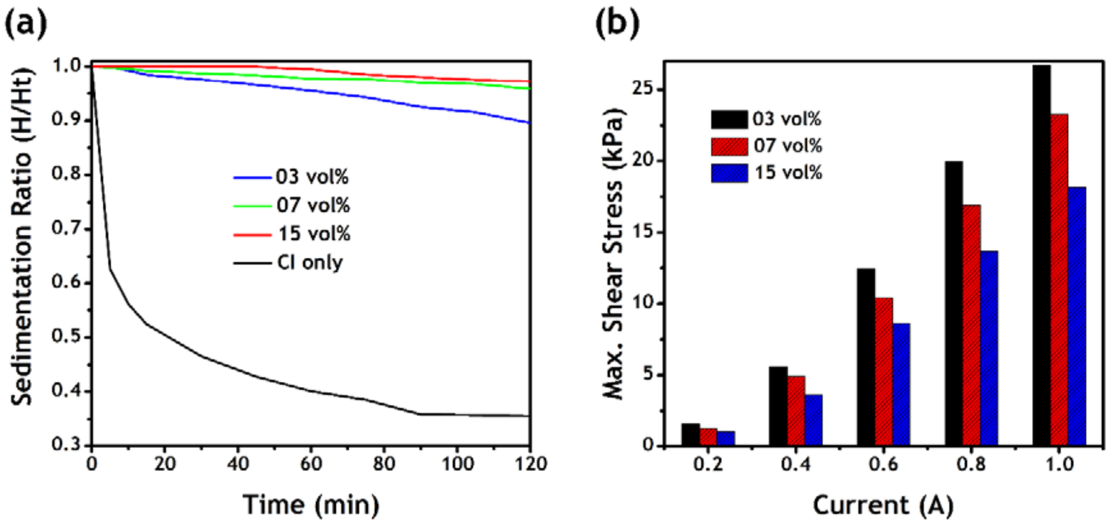

Figure S3: Rheological data for commercial MoS_2 using different volume ratio (a) Sedimentation data comparison for increasing vol%; higher vol% means more stable suspensions. (b) Shear stress data comparison for changing vol% at different applied currents. Increasing the additive ratio had a negative effect on the on-state shear stress property.
